# Comparison of microbial community assemblages in the rhizosphere of three *Amaranthus* spp.

**DOI:** 10.1371/journal.pone.0294966

**Published:** 2023-11-29

**Authors:** Joanne C. Chee-Sanford, Lynn M. Connor

**Affiliations:** USDA/ARS, Urbana, Illinois, United Station of America; University of Brescia: Universita degli Studi di Brescia, ITALY

## Abstract

Weed management remains a major challenge in cropping systems worldwide, with rising interest in ecological based approaches that can be integrated with herbicide use. Soil microbial communities may play important, yet undiscovered, roles in weed success. Little is known about the rhizosphere communities associated with weeds like *Amaranthus*, commonly known as pigweeds, and considered some of the most problematic weeds in agricultural systems. In a greenhouse experiment that allowed controlled plant growth conditions and a high number of individual plant specimens to analyze for statistical robustness (n = 8 per species), we show that specific bacterial assemblages form in the rhizospheres of *A*. *retroflexus* L. (redroot pigweed), *A*. *palmeri* S. Watson (Palmer amaranth), and *A*. *tuberculatus* (Moq.) J. D. Sauer (waterhemp). Using a relatively rapid and easy approach of T-RFLP community profiling of the 16S rRNA genes, distinct assemblages corresponded to plant species (PERMANOVA F = 14.776, p = 0.001), and further within each species, similar communities (F = 11.449, p = 0.001) were associated with three rhizosphere soil fractions taken in increasing distances away from the root tissue. These results provide the first solid basis for distinct plant-microbe relationships within three closely related *Amaranthus* species, warranting closer examination of the identities and function of the microorganisms that appear to be selectively recruited from the extant soil community. More intensive efforts to obtain the microbial taxonomic identities via sequencing are underway that can lead to further detailed studies to elucidate important functional plant-microbe interactions that may associate with weed success. Such data provides underlying key information that may ultimately exploit weed-microbe interactions in development of new integrated weed control tactics.

## Introduction

The drive toward increasing the development of integrated weed management (IWM) practices in agriculture is considered one of the grand challenges in modern cropping systems worldwide [1, 2]. Weeds continue to be the single most important biological pest to control in cropping systems, and if left unchecked, yield losses and the economic impacts can be severe. Herbicides are widely used but an overreliance on existing chemical control methods for weed management has resulted in widening occurrences of herbicide-resistant weed species at global scales. The need for new tactics that can be integrated with herbicide use have raised an increasing interest in integrated weed management (IWM) approaches that can capitalize on the ecological mechanisms that occur between weeds and their biotic/abiotic environment that could potentially lead to interferences with weed success.

*Amaranthus* species are small-seeded broadleaf annual weeds that are difficult to manage in cropping systems due to their wide distribution, extended range of seed germination times, high seed production, and long-lived seed banks [3]. Commonly referred to as pigweeds, these species are closely related and many are known to impact crop yields with rising concerns over emergence of herbicide resistances in populations [4–6]. Despite many common genotypic and phenotypic attributes, pigweed species can differ in plant size, seed production, and reproductive organ classification (monoecious vs. dioecious). Multiple *Amaranthus* species can coexist in agronomic fields, and genetic outcrossing between species is highly possible for some species in natural environments [4]. While *Amaranthus* species are important agronomic weeds of concern and significant attention has been paid to their phenotype and growth characteristics, competition with crops, and their acquisition and transmission of herbicide resistances, little information about the microbial ecology associated with these plants is available.

Microorganisms have long been known for their many important roles in ecosystem services that benefit plants, soil quality, and human health. Contemporary studies in microbial ecology are now just starting to reveal the incredible diversity present in microbial taxa and their activities [7–10], especially in soil environments where myriad physical and chemical conditions create a complex and dynamic web of biotic and abiotic interactions. While numerous studies have been conducted to examine relationships between microbes and many important plants grown widely for food and function, relatively little work has been done to assess mechanisms of interactions between weed species and native soil microorganisms, particularly those occurring in the plant rhizosphere. Microbiomes can even influence their host plasticity [11, 12], with both direct and indirect interactions possible between specific microbial taxa and host plants, or with plants growing in local proximity. Plant phenotype can influence the microbiota associated with plants [13], however these types of plant-microbe associations remain understudied. Combined with the phenotypic plasticity known among many weed species [4, 14–17] and the genetic adaptation within bacterial genomes [18–20], a better understanding of these potentially unique relationships presents an avenue of research that may advance IWM strategies.

In this study, we sought to provide a novel basis for examining microbial assemblages associated with three *Amaranthus* species considered some of the most problematic weeds in U.S. cropping systems. The microbial communities in the rhizospheres of three species, *A*. *retroflexus* L. (redroot pigweed), *A*. *palmeri* S. Watson (Palmer amaranth), and *A*. *tuberculatus* (Moq.) J. D. Sauer (waterhemp) were compared to determine if plant species formed distinct bacterial assemblages recruited from the extant populations in the soil in which they were grown. Here, we used a relatively rapid qualitative method of microbial community profiling, namely terminal restriction fragment length polymorphism (T-RFLP) following amplification of the 16S rRNA genes present in the DNA retrieved from the loose soil immediately surrounding the root zone (rhizosphere bulk soil), root-adhered soil (rhizosphere soil), and root-bound (rhizoplane) microbial communities in plants grown under a low stress greenhouse environment. The conditions imposed in the greenhouse study described here were designed to mimic summer annual cropping systems commonly seen for commercial corn and soybean production in the U.S. Midwest region, namely non-limiting N and diurnal air and soil temperature cycles similar to the period of seasonal *Amaranthus* emergence and growth. Anticipating interest in pursuit of similar research with other weed species and continued study with *Amaranthus* species, we also provide detailed methodological description to aid future molecular-based studies involving microbial community analyses.

## Materials and methods

### Plant and seed propagation

Initial seed lots of three pigweed species obtained commercially (*A*. *palmeri* and *A*. *retroflexus*, Azlin Seed Service, Leland, MS) or from plants collected around agricultural fields located in Illinois (*A*. *tuberculatus*, courtesy of N. Hausman, USDA-ARS, Urbana, IL) were used to generate seed lines for experimental use in this study. Soil used for plant growth was collected by bulk (0–10 cm depth) in Fall 2020 from the SoyFACE (Soybean Free Air Concentration Enrichment) facility (Champaign, IL), a site belonging to the University of Illinois crop research program freely accessible to university researchers and their collaborators. The SoyFACE (SF) soil was sieved (4 mm) to remove large debris and plant material, mixed by hand to homogenize, and stored at 25 °C prior to use. General soil characteristics are described in S1 Table.

To obtain experimental seed lots, each *Amaranthus* species was grown independently to avoid propagation of genetic hybrids between species. Soil (~2 kg) was placed in 14 cm^2^ polypropylene pots and 20 seeds of *Amaranthus* species were sprinkled on the surface and lightly misted with distilled (DI) water, then pressed lightly into soil but not covering. DI water (50–75 ml) was used to evenly moisten soil before placing into a controlled environment growth chamber (Conviron) programmed to cycle 16 h day length at 28 °C and 8 h night length at 22 °C, with plant watering occurring when needed. Soil moisture varied in accordance to natural cycles of wetting and drainage under the temperature conditions of the incubation chamber or daily greenhouse environment. Emergence of seedlings fully occurred after 7 d, and at 28 d plants were thinned to four plants per pot and transferred to a greenhouse (15 h supplemental lighting, day temperature range 24–27 °C, night temperature range 21–24 °C for continued growth to maturity. Plants were watered twice daily and soils were allowed to drain unrestricted through the bottom of the pots after each watering. Healthy plants underwent a nitrogen (N) fertilization regimen starting at 38 d and consisted of twice weekly addition (10 ml) of a mixture of urea (26.7 mM) and NH_4_NO_3_ (20.0 mM). No additional amendments were needed (e.g. P, K, Mg) as the ambient soil concentrations were considered adequate for plant growth (S1 Table). Plants were grown to maturity at ~85 d after germination. Distinctions between male and female plants of *A*. *palmeri* and *A*. *tuberculatus* were identifiable after 78 d. These growth conditions were henceforth established as the standard protocol for propagation of plants in experimental protocols used for rhizosphere studies. Plants used for seed lots were harvested after reaching senescence with seeds collected by hand before dry storage at 25 °C prior to any experimental use.

### Experimental set up

All *Amaranthus* species were propagated for rhizosphere specimen collection as described above except seedlings were thinned to one plant per pot to simulate conditions where plants are grown to minimize stress conditions attributed to factors such as competition for nutrients, moisture, direct antagonistic plant interactions, variable soil temperature, and shading. The relative soil moisture in each pot was monitored routinely using a soil moisture probe (Soil Master, Mosser Lee). On average, soil moisture by gravimetric measurement was maintained between 15–17% (w/w). Eight individual plants served as replicate units per plant species, with nine plant-free pots in total used as control replicates. Plants were grown to maturity and then harvested to obtain rhizosphere fractions as described below. Homogenized SF soil used for plant growth also served as the reservoir for microbial recruitment to the plant rhizosphere and seven samples in total taken from the bulk homogenate were used to represent the baseline microbial community characteristics.

### Plant rhizosphere sampling

Following daily watering and drainage on the day of sampling, plants were carefully removed from pots, allowing loose soil to fall free. Soil moisture was measured gravimetrically using ~5 g soil taken from the loose soil volume. Following, three rhizosphere fractions were separated in sequence from each plant harvested and defined as follows: 1) rhizosphere bulk (RB) soil–soil that remained loosely root bound after plant was pulled from the pot, followed by 2) rhizosphere (R) soil–soil removed from roots following mechanical shaking using an orbital shaker (300 rpm, 10 min at room temperature) into 150 ml phosphate buffered saline (PBS, pH 8), and finally, 3) rhizoplane (RP) sample–soil and associated biomass bound tightly to root surfaces removed during 30 s sonication (Bransonic ultrasonicator) in 150 ml PBS buffer containing TWEEN 80 surfactant (0.01% v/v). RB soil was removed by placing the root mass on a sterile metal tray and loose soil was carefully removed from the root mass using a sterile metal spatula and transferred to sterile plastic bags. A subsample of RB soil (0.5 g dry wt) was directly transferred into 2.0 ml bead tubes (Lysing Matrix Tube E, MP Biomedicals) containing (800 μl) to use for DNA extraction (see below) and the remaining RB soil was stored frozen (-20 °C). The remaining root mass was transferred to a 250 ml flask containing 150 ml PBS buffer followed by gentle shaking for10 min at 300 rpm using an orbital shaker for recovery of R soil fractions. Each R soil suspension was centrifuged (3220 x g) at 4 °C for 10 min, the supernatant was removed, and the soil pellet was resuspended in PBS buffer (800 μl) before transfer to 2.0 ml bead tubes. Following, each RP fraction was recovered by placing the root mass into a fresh flask containing PBS buffer and TWEEN 80 and shaken for10 min at 300 rpm on an orbital shaker before undergoing 30 s in a sonication bath. Each RP suspension was centrifuged (3220 x g) at 4 °C for 10 min, the supernatant was removed, and the pellet was resuspended in PBS buffer (800 μl), and transferred to 2.0 ml bead tubes. Following each plant growth phase, parallel-run plant-free control soils were mixed thoroughly by hand using sterile spatulas while in pots to obtain a representative homogenate of soil before subsamples of ~0.5 g (dry wt.) were removed into 2 ml bead tubes containing PBS buffer. Replicates of field SF soil used for plant propagation was sampled (~0.5 g dry wt.) and frozen on day 1 of the experimental set up. An additional attribute for each of the rhizosphere fractions obtained from *A*. *palmeri* and *A*. *tuberculatus* included distinction of male and female plants among the eight replicates for each species. For *A*. *palmeri* male (n = 3) and female (n = 5), and from *A*. *tuberculatus* male (n = 4) and female (n = 4).

### Nucleic acid extraction

The extraction method was developed in-house by modification of standard phenol-chloroform extraction methods optimized here for recovery of both DNA and RNA and detailed as follows. Only DNA was targeted for analyses in this experiment; for future reference, RNA fractions can be recovered from the nucleic acid pool following standard procedures for DNA removal and used for transcript-based analyses if needed. Each soil or rhizosphere fraction that was collected into a 2.0 ml bead tube containing PBS buffer was washed by brief mixing on a vortex mixer (Vortex Genie 2, Fisher Scientific), then centrifuged (2500 x g) for 5 min at 4 °C and the supernatant was removed. Cell lysis and separation of nucleic acids was performed by the addition of 10 μl β-mercaptoethanol (Sigma), 0.5 ml phosphate buffer (120 mM stock, pH 8), and 0.5 ml phenol-chloroform-isoamyl alcohol (25:24:1) (pH 8.0) (Sigma) to the cell pellet, followed by 2 min. of agitation at room temperature using a Disruptor Genie apparatus (Scientific Industries), then centrifuged (15,000 x g, 5 min). The supernatant was transferred to a clean 2 ml microfuge tube, avoiding volume at the phase interface. A volume of NaCl (5 mM stock) was added to obtain a final concentration of 0.7 M, mixed gently by inverting the tube several times, followed by addition of 0.1 volume of cetyltrimethyl ammonium bromide (CTAB) (10% w/v in 0.7 M NaCl, prewarmed at 65 °C), then mixed together by inverting tube several times. An equal volume of chloroform-isoamyl alcohol (24:1) was added and mixed gently by inverting tubes several times before mixture was centrifuged (15,000 x g) briefly enough to achieve phase separation. The supernatant was then transferred to a clean 2 ml microfuge tube and the chloroform-isoamyl alcohol clean-up step was repeated again, except the supernatant was transferred the second time into a clean 2 ml microfuge tube containing 1 μl glycogen (20 mg ml^-1^ stock). The nucleic acids were precipitated by addition of 1 volume polyethylene glycol (PEG) 6000 (stock 20% PEG 6000 made in 2 M NaCl). The nucleic acids were allowed to precipitate for 2 h at -20 °C, followed by centrifugation (18,000 x g, 4 °C) for 15 min. The supernatant was discarded and the pellet was washed using 200 μl ice cold ethanol (70% stock), then centrifuged (18,000 x g, 4 °C) for 5 min. The ethanol was removed by pipet and the pellet was air dried before resuspending in 50–200 μl nuclease-free water. The crude nucleic acid extract was transferred to a clean 0.5 ml microfuge tube and stored at -20 °C until further use. One sample was selected randomly to provide three technical replicates for an internal assessment of variability due to extraction efficiencies and subsequent PCR gene amplification for the soil type used in this study.

### T-RFLP community profiling based on amplification of 16S rRNA genes

The 16S rRNA genes in the DNA pools were amplified using primer set F27/1492R [21]. The forward primer F27 (5’-AGA GTT TGA TCM TGG CTC AG-3’) was labeled at the 5’ end with 6-carboxyfluorescein (6-FAM) and when coupled to the reverse primer 1492 (5’-GGT TAC CTT GTT ACG ACT T-3’), amplifies the 16S rRNA genes from a broad diversity of bacteria. The primers were HPLC-purified and obtained from IDT (Integrated DNA Technologies, Skokie, IL, USA). Stock concentrations (100 μM) of each primer were made by adding Invitrogen^™^ UltraPure^™^ DNase/RNase-Free Distilled Water (Thermo Fisher Scientific Waltham, MA, USA) and subsequently diluted for use in PCR accordingly for amplification of the target gene. PCR reactions were performed in 50 μl volumes using the Takara ExTaq PCR kit (Clontech) and a MJ Research PTC-200 Gradient Thermal Cycler. The optimized reaction mixture was the following: 1X PCR buffer, 0.2 mM each deoxynucleoside triphosphate (dNTPs), 0.025U μL^-1^ TaKaRa Ex Taq DNA polymerase, 1.0 μM each forward and reverse primers, and ~1 ng DNA. T4 gene protein 32 (New England Biolabs, 10 mg/ml stock) was added to the reaction mixture to obtain a final concentration of 62.5 μg mL^-1^. The following thermocycling program was used: initial denaturation step at 94°C for 5 min, followed by 25 cycles of 94 °C for 90 sec, 55 °C 90 sec, and 72 °C for 90 sec, then a final extension at 72 °C for 10 min. PCR products were resolved by gel electrophoresis in 2.5% High Resolution Agarose (fragments < 1 Kb) (Gold Biotechnology, Olivette, MO, USA) at 4 V cm^-1^ for 70 minutes or 1% Seakem LE Agarose (fragments > 1 Kb) (Lonza) at 4 V cm^-1^ for 50 minutes in 1X TBE buffer on a HU13 Midi horizontal gel unit (Scie-plas Ltd., Cambridge, UK). DNA ladders consisted of 10 μL of Quick-Load Purple 1kb DNA Ladder (0.1–10.0 kb) (New England Biolabs Inc., Ipswich, MA, USA) or 5 ul of 1kb DNA ladder (Gold Biotech). The PCR amplified mixture was purified further to remove excess primers and salts using the QIAquick PCR Purification Kit (Qiagen) following manufacturer’s instructions except 30 ul of nucleic acids-free water was used to elute the DNA from the column and the final DNA suspension was stored at -20 °C until restriction enzyme digestion.

The amplified 16S rRNA gene products were digested using the restriction enzyme HaeIII, previously determined to be the most optimal for generating a wide range of terminal fragments (TRF) sizes generated following enzyme digestion of the amplicon pools typically resulting from local Illinois soils. Additional enzyme candidates for digestion include HhaI and HpaII and can be used instead of HaeIII. HaeIII digestion was performed for 12 h at 37 °C using the NEB restriction enzyme kit (New England Biolabs) according to manufacturer’s instructions. Digested fragments were visualized using 2.5% high resolution agarose gel electrophoresis along with reference DNA containing fragment sizes ≤ 1 kb (Quick-Load^®^Purple 1 kb Plus DNA Ladder). Terminal fragment size analysis was performed using services available at the Roy J. Carver Biotechnology Center, University of Illinois, Urbana, IL.

### Data analyses

The TRF fragments generated from each sample were analyzed in Genemapper v. 3.7 (Applied Biosystems), calibrated against size standards 50–1000 bp (ROX1000). Data was sorted according to size after removal of fragments <50 bp and >1000 bp fragments (outside the range of calibration), and a threshold <80 relative fluorescence intensity was determined as background fluorescence signals based on analysis of water samples used as blank controls. The resulting dataset were unique patterns (i.e. T-RFLP profiles) comprised of TRFs that represented the bacterial 16S rRNA gene pool amplified from DNA recovered from each sample. The technical replicates of one sample used for internal assessment of extraction and amplification variability yielded identical T-RFLP patterns.

To assess whether the type of plant species and their rhizosphere fractions correspond to statistically different microbial communities as measured by 16S rRNA gene-based T-RFLP profiles, permutational non-parametric MANOVA (PERMANOVA) was used to assess significant differences using the PERMANOVA+ package in PRIMER v.6 software (PRIMER-E Ltd, Plymouth, UK). The PERMANOVA routine was performed using permutation of residuals under a reduced model and tests were based on a Jaccard (metric Bray-Curtis) community dissimilarity metric calculated from the relative abundance values of the T-RFLP fragments following square root transformation of the values. Statistical significance was set at p-value ≤ 0.05. Pair-wise tests were performed *a posteriori* within each fixed factor or combination of factors (i.e. plant species type, rhizosphere fraction, sample type) following PERMANOVA results. Principal coordinate analysis (PCO) based on Bray-Curtis dissimilarities was used to visualize the community assemblages. This analysis provides a pseudo F-value, based on 999 permutations, that quantifies the deviance from the null-hypothesis where large values of F correspond to large changes in community composition accordingly to the experimental factor (or combination of factors) defined for the analysis.

## Results

The total time required to attain plant maturity starting from seed germination was ~78 d for all three species of *Amaranthus* given the greenhouse growth conditions used in our experimental protocol. There was variability in the resulting plant height within each of the replicate sets established for each plant species, with overall shortest among *A*. *retroflexus* (n = 8, M = 80.32 cm, SD = 12.21) followed by *A*. *tuberculatus* (n = 8, M = 103.5 cm, SD = 31.01) and *A*. *palmeri* (n = 8, M = 154.9 cm, SD = 37.38). Within the dioecious species, the *A*. *palmeri* female plants (n = 5, M = 180.8 cm, SD = 8.308) on average were taller than the male plants (n = 3, M = 111.7 cm, SD = 16.66), while the *A*. *tuberculatus* females (n = 4, M = 102.9 cm, SD = 43.53) were on average similar in height to male plants (n = 4, M = 104.1 cm, SD = 18.67). Most female plants had mature seeds, but lacking in one female plant replicate of *A*. *tuberculatus* and one replicate of *A*. *retroflexus*.

The T-RFLP microbial community profile of each sample comprise unique size fragments (TRFs) that represent the most dominant taxa among the complex diversity of soil microbial populations typically present in varying abundances. The average number of TRFs obtained from the soil used for plant growth (SF) taken prior to the start of the experiment and considered the baseline source of microbial recruitment by the plant was n = 96, in contrast to an average n = 81 in plant-free soil controls taken at the end of the experiment. Similar numbers of TRFs ranging from n = 123–136 in the rhizosphere bulk soils (RB) and rhizosphere soils (R) were obtained from *A*. *palmeri* and *A*. *retroflexus* with fewer unique fragments (n = 103–110) present in the rhizoplanes of these two plant species (S1 Fig). The numbers of TRFs in the rhizosphere fractions taken from *A*. *tuberculatus* was generally lower overall than *A*. *palmeri* and *A*. *retroflexus* ranging from n = 80 in RB soils and in the rhizoplane, and n = 91 in the associated R soils. Variability typical to nucleic acid-based methodologies used in analyses involving complex matrices such as soil was evident in the few outlying samples and larger tails of distribution present in both types of plant-free soils (Control and SF).

**Fig 1:**
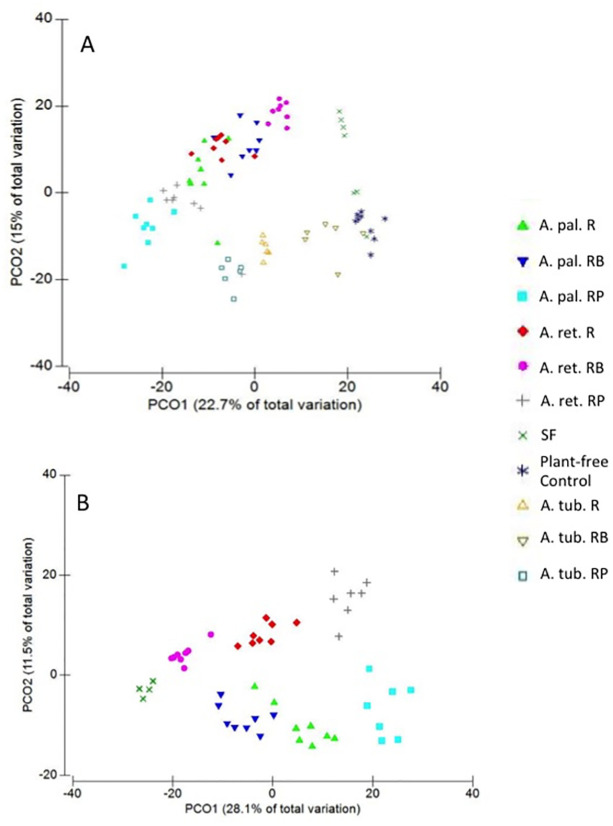
Ordination plots using principal coordinates analysis (PCO) of Bray-Curtis dissimilarities calculated using T-RFLP profiles of amplified 16S rRNA genes to compare microbial assemblages in the rhizosphere fractions (RB = Rhizosphere bulk soil, R = Rhizosphere soil, RP = Rhizoplane) of A) three plant species *A*. *palmeri (*A. pal.), *A*. *retroflexus (*A. ret.*)*, and *A*. *tuberculatus (*A. tub.*)*, and B) between *A*. *palmeri* (A. pal.) *and A*. *retroflexus (A*. *ret*.*)*. SF = soil used for plant growth. Plant-free controls were DNA from soils incubated in parallel with plants during the experiment.

The microbial communities as represented by T-RFLP profiles of amplified 16S rRNA genes showed distinct groups corresponding to the rhizosphere fractions of the three *Amaranthus* plant species (Fig 1). These rhizosphere communities were further distinguished from the originating soil community (SF) and from plant-free soil incubated under parallel conditions with experimental plant units. Results of the multivariate permutation ANOVA (PERMANOVA) based on the Bray-Curtis measure of community dissimilarities revealed the type of plant species significantly affected the community composition correspondingly within each rhizosphere fraction (F = 14.776, p = 0.001) (Table 1). The community group distinctions were greater when comparing rhizosphere fractions from *A*. *tuberculatus* to either *A*. *palmeri* or *A*. *retroflexus*, especially in the two types of soil fractions (Table 2). While the communities corresponding to each rhizosphere fractions were less differentiated between *A*. *palmeri* and *A*. *retroflexus* when compared together with *A*. *tuberculatus* (Fig 1A), there was a statistically significant effect of plant species type, further visualized in PCO ordination mapping of the communities when analyzed separately from *A*. *tuberculatus* (Fig 1B).

**Table 1:** Results of permutation ANOVA (PERMANOVA) of Bray-Curtis dissimilarities calculated between microbial community assemblages based on 16S rDNA T-RFLP profiling in the rhizospheres of three *Amaranthus* species.

*Factor*	*df*	*SS*	*MS*	*Pseudo-F*	*P(perm)*	*Unique Perms*
Plant species (Ps)	2	14190	7094.8	14.776	**0.001**	**996**
Rhizosphere fraction* (Rf)	2	10994	5497.1	11.449	**0.001**	**998**
Ps x Rf	4	6210.7	1552.7	3.2338	**0.001**	**999**
Residuals	71	34090	480.14			
Total	81	80565				

Abbreviations: *df*, degree of freedom; *SS*, sum of squares; *MS*, mean square error.

*Rhizosphere bulk soil, rhizosphere, rhizoplane samples, n = 8 plant sample individuals per *Amaranthus* species.

**Table 2:** Pair-wise comparisons between 16S rDNA T-RFLP-based communities where factor-level statistical significance was indicated in PERMANOVA results (statistically significant values in bold at p ≤ 0.05).

*Paired Groups*	*t*	*P(perm)*	*Unique Perms*
Within Rhizosphere bulk (RB) soil:			
A. pal., A. ret.	2.3936	**0.001**	927
A. pal., A. tub.	2.8245	**0.001**	858
A. ret., A. tub.	3.5319	**0.001**	852
Within Rhizosphere (R) soil:			
A. pal., A. ret.	2.1953	**0.001**	929
A. pal., A. tub.	2.7327	**0.003**	861
A. ret., A. tub.	3.5697	**0.002**	846
Within Rhizoplane (RP):			
A. pal., A. ret.	1.8658	**0.001**	933
A. pal., A. tub.	2.5781	**0.001**	861
A. ret., A. tub.	2.5596	**0.002**	854

The microbial communities also clustered in accordance with the type of rhizosphere fraction (F = 11.449, p = 0.001) (Table 1). Interestingly, the communities followed a gradient of clusters starting from the baseline microbial recruitment soil pool (SF) changing in composition as proximity to the plant root increased (rhizosphere bulk soil (RB) → rhizosphere soil (R) → rhizosplane (RP) (Fig 1). Pair-wise comparisons of the communities within each rhizosphere fraction suggested the soil-related fractions, RB and R, were more distinct with respect to plant species than those found in the rhizoplane in accordance with plant species (Table 2).

To analyze significant differences in the variability within the groups based on the replicate samples analyzed for each rhizosphere fraction, a distance-based test for homogeneity of multivariate dispersions measuring the deviation from the group centroid revealed differences between the type of rhizosphere compartment (n = 11 groups including plant-free control, n = 6–9 replicates per group) for all three plant species (df1 = 10, df2 = 71, F = 6.4528, p = 0.001). In contrast to *A*. *tuberculatus*, the communities within the rhizoplane (RP) fractions of *A*. *palmeri* and *A*. *retroflexus* demonstrated higher dispersion than their corresponding rhizosphere soil fractions (RB and R). The result suggests beta diversity does drive to some extent the differences between rhizosphere sample type.

## Discussion

The present study provides a statistically robust qualitative analysis of microbial community differences in three species of *Amaranthus* that appear to be driven by the type of plant species and the distance in relative proximity to the plant root surface. To the best of our knowledge, the results present novel information that demonstrates a solid basis for continued detailed explorations regarding the general nature of the microbial communities found in association with the root surfaces and surrounding soil zones associated with weedy plants. Despite the close genetic relatedness of the three plant species chosen for study, the distinct differences in rhizosphere zones suggest important plant-microbe relationships specific to plant species that may be critical to the underlying mechanisms that drive microbial assemblages to form around the root and how these microbes may impact the success of these and other weed species. The molecular-based community profiling afforded by techniques such as T-RFLP analysis, while unable to provide taxonomic identification, allows a relatively rapid and inexpensive approach to compare a subset of members within the microbial community likely to be the responsive populations and present in the highest abundances. Importantly, such a methodological approach can be designed with high replication of specimens for maximum statistical power before investing in significantly more costly and time-consuming microbial analyses (e.g. high throughput sequencing of amplicons or metagenomic analyses) that become potentially prohibitive with large number of samples to analyze.

The design of this experiment compared three *Amaranthus* species, predicated on the expectation that differences in phenotype and growth characteristics known among this plant genus might manifest rhizobiomes that also differ between the species. When all three species of *Amaranthus* were compared together, the results suggest that the microbiomes of *A*. *palmeri* and *A*. *retroflexus* appeared to be more similar to each other than *A*. *tuberculatus* with respect to their corresponding rhizosphere zones (Fig 1A). The distinction between the rhizobiomes of *A*. *palmeri* and *A*. *retroflexus* is more apparent when just comparing the two species apart from *A*. *tuberculatus* (Fig 1B). Such differences suggest factors specific to the species of *Amaranthus* is further driving the formation of their respective rhizobiomes. Plant species appears to largely drive the structure of the microbiome in the root zone, where the microbial community structures as represented by the T-RFLP profiles, are more similar between independently grown replicates of a given species and are further distinct between plant species demonstrate plant type directly influences microbial formation around its root. Even as proximity decreases away from the root zone, there are still distinct communities that are distinguishable from the background soil community. Key to this particular experiment was the intent to introduce a nominally identical microbial pool within the homogenized soil matrix from which each plant independently recruited its own rhizobiome. While the overall microbial assemblages were distinct between the rhizosphere fractions given the respective plant species, the microbial communities were not identical, further suggesting that other factors beyond the immediate plant root are also driving the microbial assemblages in their respective compartments as distances increase away from the rhizoplane. There were no significant differences between male and female plants of *A*. *palmeri* and *A*. *tuberculatus*.

The identification of the specific plant-related factors that appear to drive the formation of the rhizobiome compartments remain speculative, however root exudates are likely candidates to influence microbial growth. Microbial responses to root exudates are well known, among them providing a source of nutrients and cofactors, along with various mechanisms of interactions that can be beneficial to both plant and microbes [22–24]. Chemotactic responses toward the root zone of amaranths may well be in play for at least some of the microbial populations that are free living and motile in soil environments. Targeted recruitment of microbes from the soil pool may be an ecological strategy for a weedy plant to increase its survival in its environment. For example, there may be general nutritional benefits to the plant such as increasing the pool of plant-assimilatable forms of nitrogen (e.g. NH_4_^+^ or NO_3_^-^) in localized zones around the individual plant that can be mediated by such microbial processes like nitrogen fixation and ammonia oxidation. Other functions occurring within the rhizobiome may include roles of herbicidal detoxification [25, 26], plant pathogen suppression [27, 28], and plant growth promotion [29, 30]. Cellular housekeeping functions may also contribute to mechanisms leading to secondary roles for plant survival. For example, cleavage of the carbon-phosphorus (C-P) bond by the enzymatic function of C-P lyase, encoded by the structural gene *phnJ*, is harbored in many known bacterial species and generally an activity used to gain P for cell metabolism. This enzymatic activity may also be a key to glyphosate resistance, a trait that is emerging with higher occurrences in *Amaranthus* species [4]. Further regarding P as an essential nutrient for plant growth, increasing the efficiency of P absorption may allow some *Amaranthus* species to gain a competitive advantage over crops [31]. While the average phosphorus level in the soil used in this study is considered moderately high (29 ppm-P), such competitive strategies may be more critical in agronomic and other soil ecosystems where P may be limiting.

We note here that the spatial zone traditionally considered as the rhizosphere can reportedly vary from fractions of a millimeter to several centimeters away from the root edge. Defining the rhizosphere *sensu stricto* largely depends on several factors such as plant species, the type of root exudates, and the environmental factors that influence the mobility of these compounds [32]. The partitioning of the rhizosphere zone, as we defined for this study, was specifically intended to provide a standard methodology that would allow comparative analyses between different plant species with differing root size and architecture. While the rhizosphere bulk soil (RB), defined here for our purpose as being the loosely associated soil that largely remained on the root mass following plant removal, may arguably be considered non rhizosphere soil, the microbial community profiles in this fraction for all three *Amaranthus* species were clearly distinct from those in the background- and plant-free control soils (Fig 1). Future studies will necessarily establish a well defined spatial definition of the rhizosphere zones pertaining to a particular plant species in their growth environment.

Besides the direct response to root exudates, indirect effects on microbial selection in the rhizosphere zone could be mediated by other biotic factors that can ultimately affect competitive interactions between plants. For example, buckwheat (*Fagopyrum esculentum*) suppresses redroot pigweed (*A*. *retroflexus*) by changing its root exudates when sensing the presence of pigweed [33], and one could hypothesize that harboring microbial populations in the root zone that gain an advantage from these exudates (e.g. microbial carbon resource) could consequently reduce the allelopathic effect of an antagonistic plant competitor. Such an ecological strategy may be significant in areas harboring multispecies weed communities. Other indirect effects resulting in microbial recruitment may involve selection for microbial functional populations that can take advantage of localized plant-related conditions. One example of such conditions is the accumulation of nitrite in redroot pigweeds [34, 35], where nitrite-oxidizing bacteria may colonize roots to gain a growth advantage, whereby the nitrate produced may yield a nutritional advantage for the plant. Alternatively, under more reduced (i.e. anoxic) soil redox conditions, species of nitrite-reducing bacteria recruited into the rhizobiome may produce ammonium via the microbial process of dissimilatory nitrate reduction to ammonia, or DNRA, effectively changing the N-pool in localized root zones.

Interestingly, even in spatial zones more distal to the root surface like the RB soil compartment defined here, plant species also appears to explain microbial community differences. Even with the constraints imposed by the container volume as in the greenhouse experiment described here, the nature of the plant’s chemical influence more distal to the root itself still resulted in distinct differences between the rhizosphere compartments. In natural environments, the influence of the root exudates is likely to be more diffusive further away from the roots. Further, the influence of other plant cohorts or plants of different species likely to be present in natural ecosystems may also influence the natural outcome of microbial recruitment into the rhizobiome that may otherwise not occur as individual plants grown separately under the low stress conditions imposed here. The notion that in a real crop system, competition among plants for nutrients, water, and sunlight could influence the structure of the rhizobiome in manners as yet ill defined for weedy plant species. Experiments to observe the recruitment of rhizosphere communities by plants growing under higher stress conditions will shed light on whether microbial assemblages recur despite environmental conditions, however in plants undergoing higher stress, perhaps the outcome may be more strongly driven by environmental factors *in situ* affecting both plant and microbial populations.

Finally, the objective of characterizing the rhizobiomes of three closely related *Amaranthus* is of high interest in directing questions specifically geared toward understanding mechanisms of interactions of plants with extant soil microbial communities in enabling, at least in part, the nature of plant weediness. These potential mechanisms, acting alone or in synergistic fashion, may be key to better plant survival and competitiveness. Given the high microbial diversity and functional redundancy (i.e. similar function mediated by different microbial taxa) already known to be present in soil environments, the rhizobiome recruitment by the plant may still be driven toward establishing recurring underlying microbial functional characteristics, but be associated with a different set of microbial taxa. Consequently, while the resulting rhizobiomes may significantly differ in taxonomic structure between *Amaranthus* species, recruitment of functionally similar communities may be a form of generalized strategy in genetically related plants. While this study reports the efforts on bacterial (and archaeal) communities, fungi are also important members of the microbiome with many well studied interactions known to occur between plants and fungi. For example, AMF fungi are well known for enhancing plant growth [36, 37]. To address this, preliminary work using a similar molecular-based community profiling method that targets the internal transcribed spacer (ITS) region to assess fungal community patterns in *Amaranthus* rhizospheres did not reveal distinction in the fungal community structure according to plant species, however, it is possible that using a higher resolution method to obtain fungal taxonomic identities in the future may eventually reveal dominating fungal taxa in the rhizobiome not previously described in plant interactions.

## Conclusions

We conclude that plant species-specific rhizosphere communities formed among the three *Amaranthus* species used in this study, and the recruitment of microbial populations does not appear to be largely stochastic in nature. This work provides a solid basis to support the continuing pursuit of identifying the microbial taxa and potential functional genes using technically more complex methodologies such as high throughput sequencing that may include amplicons generated from targeted ribosomal- and functional genes of interest or in metagenomic or other -omics analyses. Such approaches are both labor intensive and more costly, and employment of practical designs will be needed to necessarily reduce the number of specimen to analyze but still provide statistical robustness. This is particularly the case for future pursuit of gene expression studies, where RNA-based approaches can reveal the microbial activities that may be significant in interactions with the plant rhizosphere. More in-depth community characterization based on gene sequencing is currently underway with the same three *Amaranthus* species described here. From this study, we further advocate for the use of relatively simple and inexpensive approaches such as that afforded by methods such as T-RFLP profiling of targeted genes (e.g. 16S rRNA genes, fungal ITS, nitrogen fixation, ammonia oxidation, etc.) that can be applied to large numbers of specimens for analyses and can provide highly useful preliminary assessments of community patterns for comparative studies under different plant growth conditions. The importance of soil microbial communities in the life strategies of weedy plants may be one key area leading to potential avenues that can be exploited in the pursuit of ecologically sustainable weed management practices in the future. Such burgeoning knowledge promises to contribute to more thorough understanding of the larger overall ecology of complex crop/weed/microbe/soil systems.

## Supporting information

S1 Table General physical and chemical characteristic of soil used in this study. (DOCX)

S1 Fig Box-Whisker plot of total number T-RFLP fragments (TRFs) generated following HaeIII digestion of amplified 16S rRNA genes in the community profiles of the corresponding rhizosphere fractions (RB = rhizosphere bulk soil, R = rhizosphere soil, RP = rhizoplane) of *A*. *palmeri* (A. pal.), *A*. *retroflexus* (A. ret.), and *A*. *tuberculatus* (A. tub.) (n = 8 each plant species). Control samples were taken from plant-free experimental units. SF = SoyFACE soil was used as the plant growth medium and represents the baseline community. The box represents the central 50% of the data with the 25^th^ and 75^th^ percentiles indicated by the lower and upper box edges, respectively, and the line representing the median plotted within each box. Whiskers show the tails of the data distribution. Filled circles depict outlier values.
(TIF)
