# Supplementary material for: Comparison of microbial community assemblages in the rhizosphere of three Amaranthus spp
Source: PLoS One. 2023 Nov 29;18(11):e0294966. doi: 10.1371/journal.pone.0294966 (PMC10686429; doi:10.1371/journal.pone.0294966)
Supplement: S1 Table — (DOCX) [file pone.0294966.s001.docx]

Suppl. Table 1. General physical and chemical characteristic of soil used in this study.

|  | Sand | Silt | Clay | Bulk Density | WHC | WHC | OM | pH | P Bray-1 Equiv | K | Mg | CEC | % Cation Saturation | | | NO_3_^-^ -N | NH_4_^+^ -N | TKN | Total Fe HClO_4_ extr. |
| --- | --- | --- | --- | --- | --- | --- | --- | --- | --- | --- | --- | --- | --- | --- | --- | --- | --- | --- | --- |
| Soil Textural Classification | % | % | % | g/cm^3^ | @1/3 Bar | @ 15 Bar | % |  | ppm-P | ppm | ppm | meq/100g | %K | %Mg | %Ca | ppm | ppm | % | ppm |
| Silty Clay Loam | 13 | 54 | 33 | 1.28 | 29.52 | 15.32 | 3.1 | 7.3 | 29 | 425 | 2300 | 15.6 | 3.3 | 22.8 | 73.9 | 15 | 5 | 0.168 | 19250 |
