# Supplementary figures and images for: Comparison of microbial community assemblages in the rhizosphere of three Amaranthus spp
Source: PLoS One. 2023 Nov 29;18(11):e0294966. doi: 10.1371/journal.pone.0294966 (PMC10686429; doi:10.1371/journal.pone.0294966)

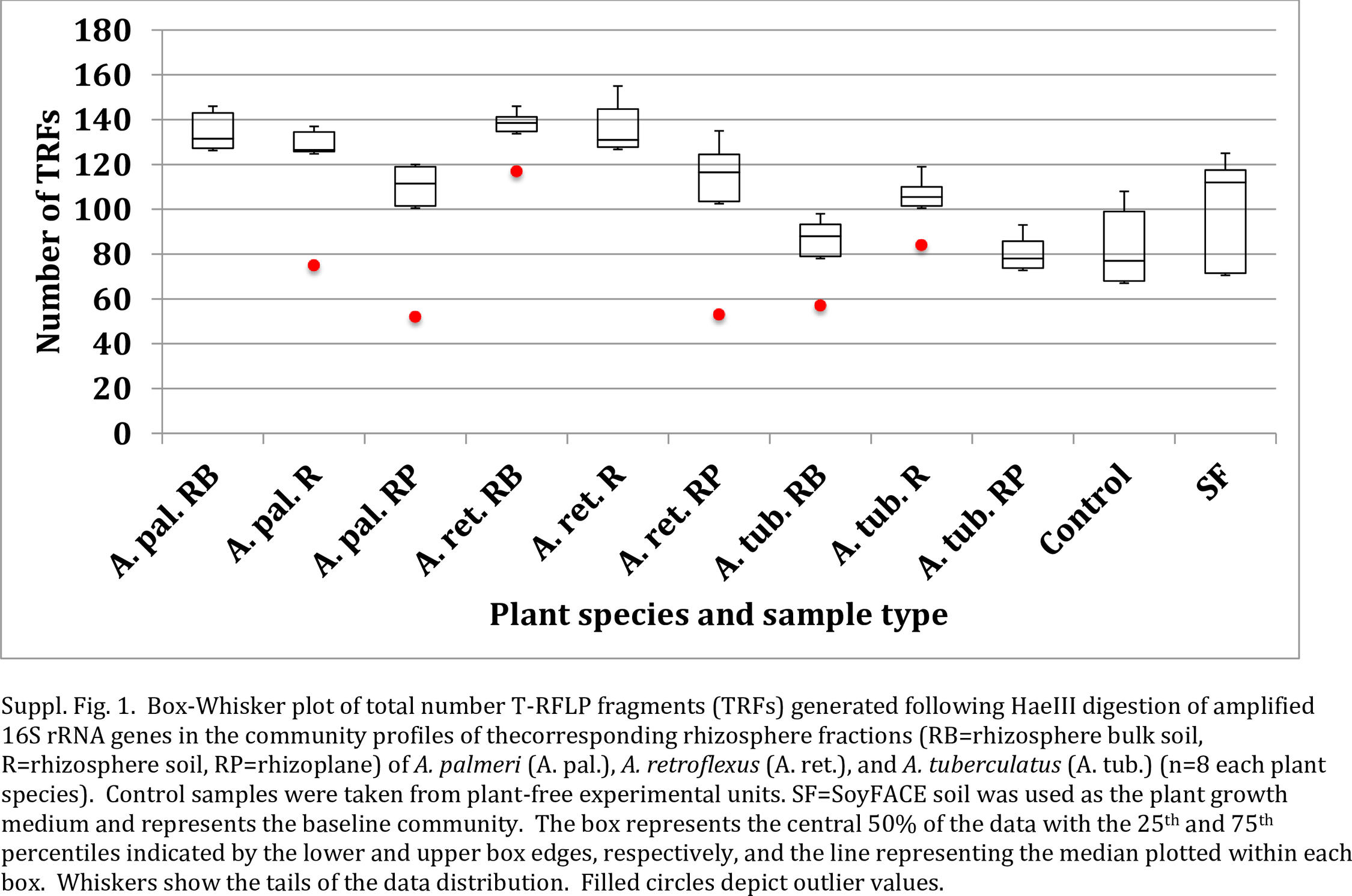

Supplement: S1 Fig — Control samples were taken from plant-free experimental units. SF = SoyFACE soil was used as the plant growth medium and represents the baseline community. The box represents the central 50% of the data with the 25th and 75th percentiles indicated by the lower and upper box edges, respectively, and the line representing the median plotted within each box. Whiskers show the tails of the data distribution. Filled circles depict outlier values. (TIF) [file pone.0294966.s002.tif]
